# The S100B Inhibitor Pentamidine Ameliorates Clinical Score and Neuropathology of Relapsing—Remitting Multiple Sclerosis Mouse Model

**DOI:** 10.3390/cells9030748

**Published:** 2020-03-18

**Authors:** Gabriele Di Sante, Susanna Amadio, Beatrice Sampaolese, Maria Elisabetta Clementi, Mariagrazia Valentini, Cinzia Volonté, Patrizia Casalbore, Francesco Ria, Fabrizio Michetti

**Affiliations:** 1 Department of Translational Medicine and Surgery, Section of General Pathology, Università Cattolica del Sacro Cuore, Largo Francesco Vito 1, 00168 Rome, Italy; gabriele.disante@unicatt.it (G.D.S.); mariagrazia.valentini@unicatt.it (M.V.); 2 Fondazione Policlinico Universitario A. Gemelli IRCCS, Largo Agostino Gemelli 1-8, 00168 Rome, Italy; 3 Cellular Neurobiology Unit, Preclinical Neuroscience, IRCCS Santa Lucia Foundation, Via del Fosso di Fiorano 65, 00143 Rome, Italy; s.amadio@hsantalucia.it (S.A.); c.volonte@hsantalucia.it (C.V.); 4 Istituto di Scienze e Tecnologie Chimiche “Giulio Natta” SCITEC-CNR, Largo Francesco Vito 1, 00168 Rome, Italy; beatrice.sampaolese@scitec.cnr.it (B.S.); elisabetta.clementi@scitec.cnr.it (M.E.C.); 5 Institute for Systems Analysis and Computer Science, IASI-CNR, Largo Francesco Vito 1, 00168 Rome, Italy; patrizia.casalbore@cnr.it; 6 Department of Neuroscience, Università Cattolica del Sacro Cuore, Largo Francesco Vito 1, 00168 Rome, Italy; 7 IRCCS San Raffaele Scientific Institute, Università Vita-Salute San Raffaele, 20132 Milan, Italy

**Keywords:** S100B, multiple sclerosis, relapsing–remitting experimental autoimmune encephalomyelitis, pentamidine

## Abstract

S100B is an astrocytic protein acting either as an intracellular regulator or an extracellular signaling molecule. A direct correlation between increased amount of S100B and demyelination and inflammatory processes has been demonstrated. The aim of this study is to investigate the possible role of a small molecule able to bind and inhibit S100B, pentamidine, in the modulation of disease progression in the relapsing–remitting experimental autoimmune encephalomyelitis mouse model of multiple sclerosis. By the daily evaluation of clinical scores and neuropathologic-molecular analysis performed in the central nervous system, we observed that pentamidine is able to delay the acute phase of the disease and to inhibit remission, resulting in an amelioration of clinical score when compared with untreated relapsing–remitting experimental autoimmune encephalomyelitis mice. Moreover, we observed a significant reduction of proinflammatory cytokines expression levels in the brains of treated versus untreated mice, in addition to a reduction of nitric oxide synthase activity. Immunohistochemistry confirmed that the inhibition of S100B was able to modify the neuropathology of the disease, reducing immune infiltrates and partially protecting the brain from the damage. Overall, our results indicate that pentamidine targeting the S100B protein is a novel potential drug to be considered for multiple sclerosis treatment.

## 1. Introduction

Multiple sclerosis (MS) is an autoimmune disease now recognized as a global disease, affecting more than 2.3 million persons worldwide, with an occurrence especially high in Western Europe and North America [1]. The pathologic hallmarks of the disease are demyelination and axonal loss, characterized by focal lesions/plaques that are scattered throughout the white and gray matter of the central nervous system (CNS). These plaques represent a combination of pathologic features, including edema, inflammation, gliosis, demyelination, and/or axonal loss. A variety of pathogenic processes have been implicated in plaque formation, including oxidative stress promoted by macrophages/microglia, neurotoxic factors secreted by activated T-cells, and autoantibodies directed at self-antigens. However, the key mechanisms in this disease are essentially unknown. Hence, the identification of such factors is still a necessary prerequisite to develop more efficacious therapeutic strategies [2].

S100B [3] is a small EF-related Ca^2+^/and Zn^2+^/binding protein, which is mainly synthesized by astrocytes, and, to a lesser extent, by oligodendrocytes in the CNS. It exerts both intracellular and extracellular actions. While a clearly defined intracellular function at present has not been delineated for this protein, secreted S100B is regarded to act as a paracrine and autocrine factor for astrocytes, with concentration-dependent effects. Under physiological conditions, astrocytes secrete S100B, which exerts a neurotrophic action in nanomolar concentration. Under stress conditions, including nervous tissue inflammation, astrocytes secrete S100B that has a neurotoxic effect at micromolar concentration, behaving as a danger/damage-associated molecular pattern (DAMP) molecule. S100B has been shown to interact with surrounding cell types mainly through the activation of the receptor for advanced glycation endproducts (RAGE), a ubiquitous, transmembrane immunoglobulin-like receptor known to act as both inflammatory intermediary and critical inducer of oxidative stress. S100B in biological fluids is regarded to be a reliable biomarker of active neural injury [4] but, more recently, evidence is accumulating that indicates that this protein plays a key role in the pathogenic processes of neural disorders for which it also acts as a biomarker, including acute brain injury, Alzheimer’s disease (AD), Parkinson’s disease (PD), and amyotrophic lateral sclerosis [5].

It is reasonable to hypothesize that high concentrations of S100B may play a promoting role in MS, based on correlative evidence. Elevated levels of S100B were detected in the cerebrospinal fluid (CSF) [6,7] and sera [7] of MS patients in the acute phase, being reduced in the stationary phase of the disease, and an increased expression of S100B has been detected in both active demyelinating and chronic active MS plaques [8]. In ex-vivo demyelinating models, a marked astrocytic elevation of S100B was observed upon demyelination, while inhibition of S100B action reduced demyelination and downregulated the expression of inflammatory molecules [7]. In addition, blockade of RAGE has been shown to suppress demyelination in a rodent demyelinating model of experimental autoimmune encephalomyelitis (EAE) [9], and, more recently, the S100B/RAGE axis has been shown to play a crucial role in oligodendrocyte myelination processes [10]. Overall, these data suggest a potential role of S100B in MS pathogenesis and, as a consequence, its suitability as a therapeutic target for the disease. However, the in vivo involvement of this molecule in MS processes has never been studied.

This work aims at investigating the effects of blocking S100B in a recognized experimental in vivo model of MS, such as the relapsing–remitting experimental autoimmune encephalomyelitis (RR–EAE) induced in Swiss Jim Lambert (SJL) mice. To this purpose, pentamidine isethionate (PTM) appears to be a suitable tool, since this antiprotozoal drug has been shown to inhibit S100B activity by blocking the interaction at the Ca^2+^/p53 site of the protein [11,12,13]. Here, we will show a clinical amelioration of disease scores in RR–EAE SJL mice after PTM administration, accompanied by coherent variations of neuropathologic/biomolecular parameters.

## 2. Materials and Methods

### 2.1. Animal Procedures

RR–EAE in the SJL mouse model: to induce active EAE, we immunized female SJL (8–10 week-old) mice, purchased from Charles River, with an emulsion composed by a fragment of the proteo-lipid protein (PLP139–151, the immunodominant epitope) and complete Freund’s adjuvant (CFA4X) containing 4 mg of heat-killed and dried Mycobacterium tuberculosis (strain H37Ra, ATTC 25177, and Bordetella Pertussis toxin (Sigma-Aldrich S.r.l., Milan, Italy). Immunization and treatments are described in the timetable in Figure S1a, according to the procedures described in our previous works [14,15,16,17]. Mice have been monitored daily for body weight, development of clinical signs and symptoms (CSS), and for disease remission/relapse. CSS have been scored using the scale described by Miller et al. [18]. In the light of the notion that blood–brain barrier (BBB) is damaged during the demyelinating disease, PTM was administered intraperitoneally, with a dosage of 4 mg/kg; SJL mice were randomly distributed into four different groups: untreated healthy controls (Ctrl), PBS–EAE group (vehicle), PTM-treated healthy controls, and PTM-treated EAE affected mice. Seven days after RR–EAE induction, the group of RR–EAE mice received a daily intraperitoneal administration of PTM (Sigma-Aldrich S.r.l., St. Louis, MO, USA) for 30 days (4 mg/kg).

Each group of mice was composed of 9 mice and the procedure was repeated in two experiments (with a total of 36 EAE affected mice, 18 treated with PTM and 18 with vehicle). Thirty-six additional SJL mice were not immunized but used as controls, 18 untreated and 18 treated with PTM). As expected from this RR–MS mouse model, 10% of mice were withdrawn from the protocol because of excessive reaction to immunization, unresponsiveness/anergy to immunization, or death for unknown causes. For ethical reasons according to the guidelines for animal wellness, mice with exaggerated symptoms were excluded and sacrificed before the foreseen timepoints (3 of the EAE group treated with vehicle and 1 of the EAE group treated with PTM). Hence, we analyzed 32 animals suffering from EAE (17 in the PTM-treated group and 15 in the vehicle-treated group). The animals were sacrificed at onset, remission, and relapse phases of the EAE (at least 5 mice for each timepoint). Specifically, mice euthanized during onset phase were chosen at the beginning of acute phase, when they reached a CSS between 2 and 3. Mice sacrificed during remission phase, were sacrificed when they presented at least 1 point of CSS below the peak reached during acute phase. On the contrary, relapse was analyzed sacrificing mice when they reached at least 0.5 point of CSS above their remission mean CSS. Ctrl, vehicle, and healthy groups were sacrificed accordingly. Mice were perfused with saline solution under deep anesthesia (87.5 mg/Kg ketamine and 12.5 mg/Kg xylazine; 0.1 mL/20g mouse wt i.p.), the brain was removed and one hemisphere was used for morphological analysis, after fixation (48 h in 4% paraformaldehyde (PFA)), while the lysates extracted from the other hemisphere have been used for molecular biology assays and processed accordingly [19].

### 2.2. RT–qPCR Assay

Total RNA was isolated with a SV Total RNA Isolation System (Promega, Madison, WI, USA) and RNA concentration was evaluated by spectrophotometric reading at 280 and 260 nm. Total RNA was used for first strand cDNA synthesis with a High-Capacity cDNA Reverse Transcription Kit (Applied Biosystems). PowerUp™ SYBR^®^ Green (Thermo Fisher Scientific, Waltham, MA, USA) Master Mix (2×) reagents were used according to the manufacturer’s recommendations. The quantification of gene expression was obtained from Applied Biosystem 7900HT Fast Realtime PCR System. PowerUp™ SYBR^®^ Green (Thermo Fisher Scientific, Waltham, MA, USA) Master Mix (2×) reagents were used according to the manufacturer’s recommendations. Primers were bought from SIGMA-Aldrich (St. Louis, MO, USA): for *S100B* and inducible nitric oxide synthase (*iNOS*) after design by NCBI Primer-Blast program (https://www.ncbi.nlm.nih.gov/tools/primer-blast), while the other oligos were deduced from literature [20,21,22,23,24]. Each gene target quantification reaction was performed separately with the respective primer sets (Table S1). Conditions were as follows: 50 °C for 2 min, followed by 95 °C for 10 min, forty cycles at 95 °C for 15 sec, followed by 60 °C for 1 min. The melt standard curve was at 95 °C for 15 s, followed by 60 °C for 1 min, 95 °C for 15 sec, and finally, 60 °C for 15 sec. Gene expression results were analyzed using Applied Biosystem software, SDS 2.4.1. Relative mRNA expression levels were calculated by normalizing examined genes against b actin using the 2^−ΔCt^ method.

### 2.3. Homogenate Preparation

Frozen brain (200 mg) was transferred to a mortar and the tissue crushed in liquid nitrogen with a pestle. During homogenization, the tissue was kept completely frozen to preserve functional and structural integrity of proteins. The samples were transferred to microcentrifuge tube with 1 mL of ProteoJET mammalian cell lysis reagent (Fermentas life Science, Waltham, MA, USA) plus a protease inhibitor cocktail, vortexed and then centrifuged at 13,000 rpm at 4 °C for 10 min. The protein concentration of supernatants was measured with Bio-Rad Protein Assay in 96-well microplates. All the samples were normalized to the same protein concentration and used according to specific experimental procedures.

### 2.4. ROS Detection

Radical oxygen species (ROS) levels were measured using the OxiSelect™ Intracellular ROS Assay Kit (Green Fluorescence; Cell Biolabs, Inc. San Diego, CA, USA) according to the manufacturer’s instructions. Briefly: 50 μL (0.5 mg/mL of protein) of each homogenate were added to a 96-well plate suitable for fluorescence measurement. Then, 50 μL of catalyst and 100 μL of 2′, 7′-dichlorodihydrofluorescin diacetate (DCFH-DA) solution were added to each well. The plate was read with a fluorescence plate reader at 480 nm excitation/530 nm emission. A standard curve was made using 2′,7′-dichlorodihydrofluorescein standard solution (manufacture’s solution).

### 2.5. NOS Activity

NOS enzyme activity was measured by colorimetric method using the NOS Activity Assay Kit (BioVision, Kampenhout, Belgium), according to the manufacturer’s indications. Briefly, 30 µL (200 µg protein) of tissue homogenates were added in a 96-well plate. Successively, 40 μL of the reaction mix (NOS cofactor 1, NOS cofactor 2, NOS substrate, and nitrate reductase) were added in each well and, after 60 min of incubation, 50 µL of Griess reagent were added in each well, forming a purple azo dye by interaction with nitrites. The optical density of this dye was measured at 540 nm using a microplate reader. To generate the calibration curve, a standard solution, corresponding to 0, 250, 500, 750, 1000 pmol/well nitrites standard was used. Nitric Oxide Synthase Specific Activity was calculated = B/TxC = mU/mg of protein, where B is nitrite amount in sample well from standard curve, T is reaction time (60 min), and C is the amount of protein.

### 2.6. S100B Protein in Brain Homogenates

The quantitative measurement of S100B protein in brain homogenates was performed using S100B in vitro SimpleStep ELISA^®^ (enzyme-linked immunosorbent assay) kit (Abcam, Cambridge, UK) according to the manufacturers’ instructions. Briefly, 50 μL (1 mg/mL protein) of homogenates or standard samples were added to appropriate wells. After the addition of 50 μL of the antibody cocktail to each well, the plate was incubated 1 h at room temperature. Finally, after series of washes, 100 μL of TMB developing solution and 100 μL of stop solution were added to each well. The plate was read at 450 nm using a microplate reader and the quantitative measurement of S100B protein in mouse homogenates was reported as absorbance at 450 nm.

### 2.7. Tissue Processing and Histology

After fixation (4% PFA) and cryoprotection in sucrose 30%, 30 µm thick serial brain emisections taken from +1.34 to +4.66 bregma coordinates according to the atlas of Paxinos and Franklin [25], and spinal cord sections were cut with a cryostat (Leica CM1860 UV, Leica Biosystems, Nussloch Germany). One out of every three sections was stained for immunofluorescence and/or immunohistochemistry analysis to detect the following antigens: CD68 (Bio-Rad/AbD Serotec, CA, US, 1:200), a marker of activated microglia, CD4^+^ (Bio-Rad/AbD Serotec, Raleigh, NC, USA 1:50) for T-cell infiltrates, glial fibrillary acidic protein (GFAP; Cell Signaling Technology, MA, US, 1:500) and S100B (Novus biological, CO, USA, 1:1000) for astrocytes, myelin basic protein, MBP (Cell Signaling Technology Denver, MA, USA, 1:200) for myelin sheaths, and NeuN (Chemicon International, CA, USA, 1:100) for neurons.

### 2.8. Immunohistochemistry

After preincubation with 0.3% H_2_O_2_ in PBS, the sections were incubated at 4°C with primary antibodies in PBS-0.3% Triton X-100, 2% normal donkey serum (NDS). Following the use of biotinylated donkey anti-mouse or donkey anti-rat antibodies (Jackson ImmunoResearch Europe Ltd., Ely, UK), avidin-biotin-peroxidase reactions were performed (Vectastain, ABC kit, Vector, Burlingame, CA, USA), using 3,3’-diaminobenzidine (Sigma-Aldrich, St. Louis, MO, USA) as a chromogen. The sections were then analyzed using an Axioskop 2 optical microscope (Zeiss, Oberkochen, Germany), with Neurolucida software (MBF Bioscience, Williston, VT, USA) for image acquisition. The quantification of the tissue area positive for CD68/CD4/GFAP was performed with the NIH ImageJ software. The areas were calculated as CD68/CD4/GFAP positive area/total area analyzed, and indicated as percentages of stained area.

### 2.9. Immunofluorescence

The sections were blocked with 10% NDS in 0.3% Triton X-100 in PBS and incubated with primary antisera/antibodies in 0.3% Triton X-100 and 2% NDS in PBS, for 24–48 h at 4 °C and processed for immunofluorescence. The sections were washed thoroughly and incubated with appropriate fluorescent-conjugated secondary antibodies for 3 h at room temperature. The secondary antibodies (Jackson Immunoresearch, Philadelphia, PA, USA) in 0.3% Triton X-100 and 2% NDS in PBS were Alexa Fluor^®^ 488-AffiniPure donkey anti-mouse IgG (1:200, green), Alexa Fluor^®^ 488-AffiniPure donkey anti-rabbit IgG (1:200, green), Cy5-conjugated donkey anti-mouse IgG (1:100, blue), Cy3-conjugated donkey anti-rat IgG (1:100, red), and Cy3-conjugated donkey anti-rabbit IgG (1:100, red). After rinsing, the sections were stained with the nucleic acid blue dye Hoechst 33,342 (1:1000), mounted on slide glasses, covered with fluoromount medium (Sigma-Aldrich, St. Louis, MO, USA) and a coverslip, and analyzed by confocal microscopy. Immunofluorescence analysis was performed by confocal laser scanning microscope (Zeiss LSM 800) equipped with four laser lines: 405, 488, 561, and 639 nm. Brightness and contrast were adjusted with the Zen software 3.0 blue edition (Zeiss, Oberkochen, Germany). For lesion and cellular infiltrates detection, Luxol fast blue and cresyl fast violet combined staining (Kluver–Barrera) was performed.

### 2.10. Statistical Analysis

Student’s *t*-test, one-way ANOVA or two-way ANOVA (with PTM-treatement as main factor) were performed to examine the effects and possible interaction of independent variables (GraphPad 6.0 software). When appropriate, post hoc comparisons were made using Tukey’s HSD, with a significance level of *p* < 0.05. For statistical analysis of quantitative PCR data, the unpaired *t*-test was used to compare ΔCt values across the replicates, setting the *p*-value cut-off at 0.05.

All experimental work has been conducted in accordance with relevant national legislation on the use of animals for research, referring to the Code of Practice for the Housing and Care of Animals Used in Scientific Procedures and the protocol was approved by the Ethics Committee of animal welfare organization (OPBA) of the “Università Cattolica del Sacro Cuore” of Rome and by the Italian Ministry of Health (authorization number 321/2017-PR, protocol number 1F295.34/04-11-2016, date of approval 12th April 2017).

## 3. Results

### 3.1. PTM Treatment Ameliorates EAE

**Figure 1:**
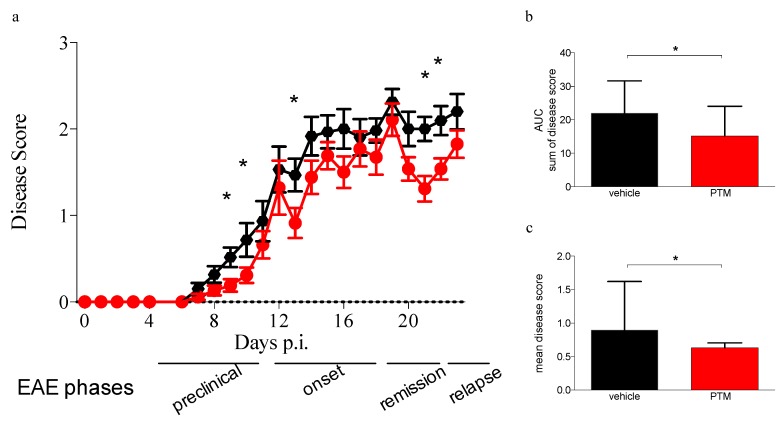
Clinical signs of neuroinflammation and demyelination of relapsing–remitting experimental autoimmune encephalomyelitis (RR–EAE) are ameliorated by pentamidine. (**a**) Clinical symptoms score (CSS) from day 1 to day 23 p.i. of 32 EAE-affected animals (4 mice were withdrawn from the experiments because of excessive reaction to immunization, unresponsiveness/anergy to immunization, or death for unknown causes), pentamidine isethionate (PTM)-treated (red) and vehicle treated (black). The circles and confidence bars represent the mean and the standard deviation of the CSS of the entire group of mice for each day p.i. Statistically significant differences of the CSS are observed at onset, during remission and at relapse of EAE (Student *t*-test: *p* = 0.02 at day 9 p.i.; *p* = 0.05 at day 10 p.i.; *p* = 0.04 at day 13 p.i.; *p* = 0.002 at day 21 p.i.; *p* = 0.01 at day 22 p.i.). At onset (days 12–15 p.i.), 10 mice (5 from each group, CSS comprised between 2 and 3 of each individual mouse) were sacrificed. Ten mice (5 from each group, at least 1 point of CSS below their individual peak CSS reached during acute phase) were sacrificed at remission (days 18–23 p.i). Finally, 12 mice (6 from each group, when they reached at least 0.5 points of CSS above their individual remission mean CSS) were sacrificed at relapse (day 30 p.i.). The average CSS values after 24 days p.i. are not shown due to the low number of mice (only 12 mice remained) and the heterogeneity among them. b and c: Mean of cumulative diseases (sum of CSS from day 1 to the day of sacrifice) of each mouse (**b**) and the mean of the diseases (sum of CSS from day 1 to the day of sacrifice divided by the number of days) of each animal (**c**). Both graphs show the two groups (with colors in concordance to figure a) displaying the overall significant impact of PTM on CSS and on the amelioration of symptoms. Statistical analysis has been performed using the Mann–Whitney test (*p* = 0.04 for b and *p* = 0.01 for c; * *p* < 0.05).

A total of 32 mice were examined for disease clinical course, immunohistochemistry for CD68 and Kluver–Barrera staining, in two independent experiments (Figure 1 and Figure S1b,c). As detected by immunohistochemistry for CD68 and Kluver–Barrera staining, significant increase in CD68 immunoreactivity with decrease in myelin content and parallel increase in cellularity could be seen in the CNS of immunized mice. Inflammatory lesions were observed with the typical periventricular infiltration and accumulation of mononuclear cells. In cerebellar sections, particularly evident demyelinating areas were highlighted by lack of MBP and NeuN immunofluorescent staining (Figure S1d). PTM-treated group showed a lower severity of symptoms at onset (*p* = 0.02 at day 9, *p* = 0.05 at day 10) and during the remission and relapse phases (*p* = 0.04 at day 13, *p* = 0.002 at day 21 and *p* = 0.01 at day 22), as evaluated by Student’s *t*-test corrected for multiple comparisons using the Holm–Sidak method. This effect can be appreciated comparing also the sums and the means of disease scores of the two groups of mice respectively with a *p* = 0.04 and a *p* = 0.01 after nonparametric Mann–Whitney test. The disease scores of untreated and PTM-treated healthy controls are not shown, as both groups did not develop any sign of disease. Thus, these data indicated that PTM is able to delay the disease and to reduce its overall severity (Figure 1a–c)

### 3.2. PTM Treatment Attenuates Neuroinflammation

To evaluate the impact of PTM on neuroinflammation, the expression of genes encoding for inflammatory cytokines during RR–EAE has been evaluated by qPCR performed on total mRNA extracted from the emi-brains of treated (EAE/PTM), untreated RR–EAE (EAE/vehicle), and CTRL mice samples (healthy PTM-treated mice).

**Figure 2:**
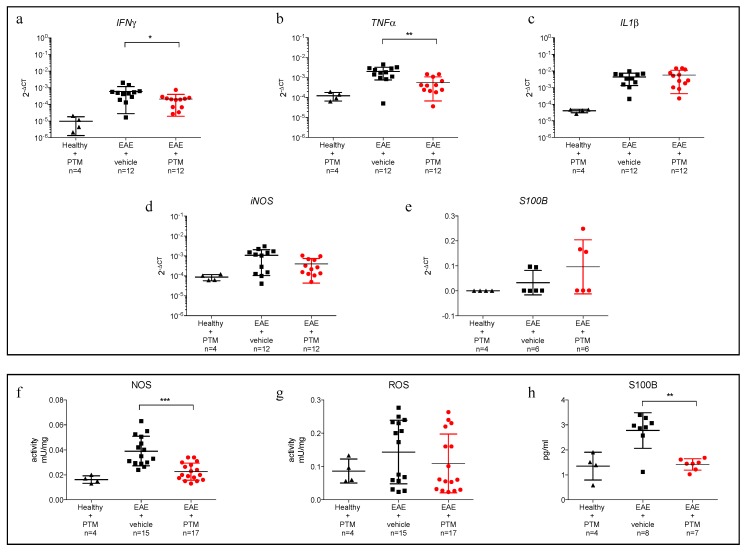
Impact of pentamidine (PTM) on gene expression and protein levels of inflammatory cytokines, S100B, nitric oxide synthase (NOS), and radical oxygen species (ROS) during EAE. (**a**–**c**) qPCR, performed on total mRNA extracted from the emi-brain of treated (PTM), untreated (vehicle) EAE, and CTRL (PTM treated healthy mice), shows a significant reduction in PTM-treated animals for the expression of *IFNγ* ((**a**), *p* = 0.03) and *TNFα* ((**b**), *p* = 0.008), despite no difference of *IL1β* (**c**). No change of inducible nitric oxide synthase *iNOS* and *S100B* mRNA is observed (**d**,**e**). Colorimetric activity assay for NOS shows a significant difference (**f,** vehicle vs. PTM *p* < 0.0001), while ROS activity does not reveal any change during PTM treatment (**g**). S100B has been measured by ELISA (h) resulting in a significant downregulation of the S100B protein (*p* = 0.0085). Statistical analysis has been performed using Mann–Whitney test. The qPCR analyses for cytokines and *iNOS* (**a**–**d**) have been performed on a total of 28 mice (12 EAE–PTM-treated, 12 EAE-vehicle-treated and 4 healthy-untreated), excluding EAE affected mice sacrificed during remission phase. The qPCR and the ELISA for S100B (**e**,**h**) were performed on mice of the second experiment (because the samples of first experiment were not usable in terms of quality and quantity) on a total of 19 mice (7 EAE–PTM-treated, 8 EAE–vehicle-treated and 4 healthy untreated); for S100B qPCR three results were missing (1 in EAE–PTM-treated and 2 EAE–vehicle-treated groups). Colorimetric activity assays for NOS and ROS (**f**,**g**) have been performed on 36 mice (17 EAE–PTM treated, 15 EAE–vehicle treated and 4 healthy untreated). For graphical reasons, *y*-axes are displayed in logarithmic for cytokines and iNOs mRNA expression (**a**–**d**) and linear *y*-axes for S100B mRNA expression (**e**) and colorimetric and ELISA assays (**f**–**h**). * *p* < 0.05; ** *p* < 0.001; *** *p* < 0.0001.

A significant decrease of neuroinflammatory parameters in PTM-treated animals was revealed by the reduction of mRNA expression for Interferon *γ*, *IFNγ* (Figure 2a, vehicle vs. PTM *p* = 0.03, Mann–Whitney test) and for tumor necrosis factor *α*, *TNFα* (Figure 2b, vehicle vs. PTM *p* = 0.003, Mann–Whitney test). We could not observe a statistically valid difference in the levels of mRNA specific for interleukin β, *IL1β* (Figure 2c).

Finally, to confirm the predictive role of S100B levels on MS pathogenesis, we measured mRNA of *iNOS* and *S100B*, finding no differences in terms of gene expression levels (Figure 2d,e). NOS are a family of enzymes that catalyze the production of nitric NO from L-arginine. NO plays an important role in neurotransmission, vascular regulation, immune response, and apoptosis. Three isoforms of NOS have been identified: two constitutive enzymes, neuronal NOS (nNOs) and endothelial NOS (eNOS), and one inducible enzyme (iNOS). Colorimetric activity assay for NOS resulted in a significant difference in vehicle vs. PTM (Figure 2f, *p* < 0.0001, Mann–Whitney test), while no changes were observed in ROS activity (Figure 2g). The quantification of S100B by ELISA (Figure 2h) resulted in a significant downregulation of S100B protein after PTM treatment (vehicle vs. PTM, *p* = 0.0085, Mann–Whitney test). As found for iNOS, the variation of S100B protein expression did not correspond to a change in terms of mRNA. To further dissect whether PTM modulates inflammatory cytokines during the relapse phase, we correlated the gene expression levels of inflammatory cytokines with the disease course (Figure S2a,b). PTM reduced the expression levels of these genes, in particular during the relapse phase.

**Figure 3:**
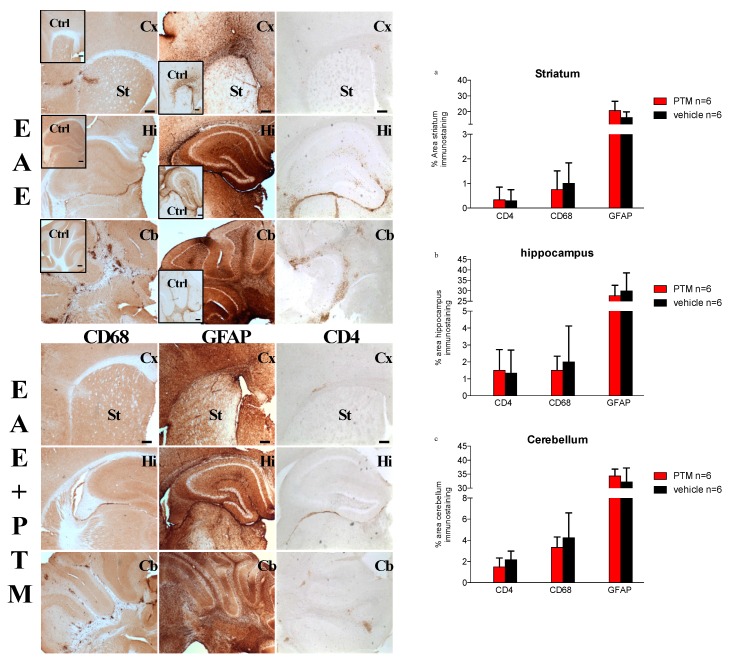
Effects of pentamidine (PTM) on immune infiltrates and glial fibrillary acidic protein (GFAP) in the central nervous system (CNS) of RR–EAE mice. EAE (*n* = 6) and EAE–PTM (*n* = 6) mice were sacrificed 30 days after immunization in the relapse/late onset of EAE (sacrificed when they reached at least 0.5 point of CSS above their individual remission mean CSS). Immunohistochemistry was performed with CD68, GFAP, and CD4 antibodies on serial brain emisections (30 μm) of control (Ctrl), EAE, and EAE–PTM mice. Cx = cortex; St = striatum; Hi = Hippocampus; Cb = cerebellum. Scale bars = 250 μm. The quantifications are displayed on the bar graph on the right of the figure (EAE–PTM-treated in red versus EAE-vehicle treated in black). Immunocytochemistry and DAB assays revealed that pentamidine seems to decrease the expression of CD68 in all the analyzed areas (**a**–**c**). CD4 infiltrates seem to be reduced by PTM in cerebellar area only (**c**), and GFAP percentages are increased in all the areas except in hippocampus (**b**). All these regional cell counts, although not significant, would indicate a different impact of the inhibition of S100B within each cerebral area.

**Figure 4:**
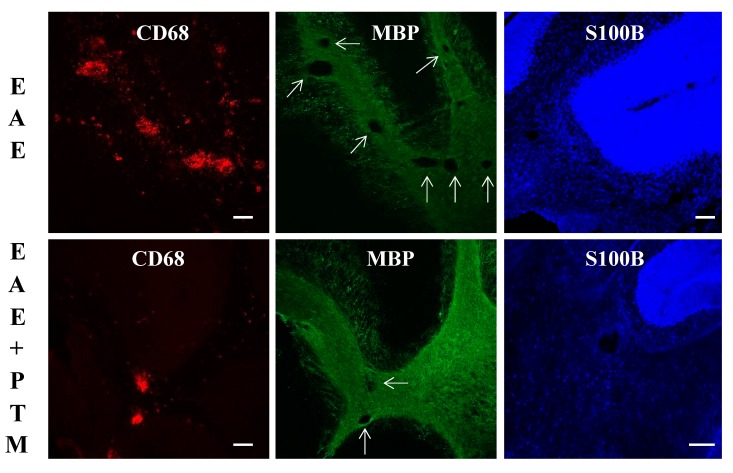
Immunofluorescence in representative cerebellar sections of EAE mice and PTM-treated EAE mice. CD68-labelled infiltrates accompanied by demyelinating lesions (arrows) are evident in EAE mice, which appear to be reduced in PTM-treated EAE mice, as shown by CD68 (red) and MBP (green) signals. Decreased S100B immunolabelling (blue) also appears in PTM-treated EAE mice. Scale bars = 100 μm.

To support our observations about clinical scores, we examined the demyelination and immune infiltrates in the CNS of RR–EAE mice treated with/without PTM, as shown by immunohistochemistry and immunofluorescence analysis (Figure 3 and Figure 4). As expected, in cerebral cortex–striatum, hippocampus, and cerebellum, the CD68 and GFAP labeling were apparently increased in EAE with respect to Ctrl mice (Figure 3 insets). Moreover, after PTM treatment, the CD68 and CD4 signals were in part decreased presenting low amount of cellular infiltrates, particularly in the cerebellum, where, however, the differences in quantification of the areas of CD68/CD4/GFAP/positive tissue, using NIH image software, did not reach statistical significance (Figure 3a–c and Figure S3). Moreover, the GFAP signal remained constant in all the analyzed brain areas (Figure 3). When examined by immunofluorescence, cerebellar sections also evidenced CD68-labelled infiltrates accompanied by demyelinating lesions in EAE mice, which appeared to be reduced in PTM-treated EAE mice. Also, S100B immunofluorescence appeared to be decreased in PTM-treated EAE mice, in accordance with S100B protein immunochemical measurements shown in Figure 2h (Figure 4).

## 4. Discussion

The present data indicate that PTM, which is regarded to block S100B protein, induces clinical disease scores amelioration accompanied by some improvement of neuropathologic and biomolecular parameters in a recognized experimental in vivo model of MS (RR–EAE in the SJL mice).

RR–EAE is a well-accepted preclinical model to study MS pathogenesis and therapy in the mouse: it shares with the human disease key immunological and pathological features, including patch inflammation, demyelination, axonal loss, and gliosis. Similarly to most common human MS manifestations (about 80%), RR–EAE in the SJL mouse is a CD4^+^ T-cell-mediated autoimmune disease directed against protein components of CNS myelin, resulting in a relapsing–remitting clinical course of paralysis [26].

S100B is regarded as a DAMP, also sharing with these molecules some characteristics such as the interaction with RAGE, the ability to stimulate microglial migration, the non-canonical secretion modality that bypasses the Golgi route [5]. In particular, the protein has been shown to induce a RAGE-dependent autocrine loop in astrocytes, which results in a pro-inflammatory phenotype [27] also stimulating IL6 and TNFα secretion [28]. In addition, S100B, which has also been reported to be expressed and secreted by CD8^+^ T- and NK-cells on stimulation [29], upregulates cyclooxygenase-2 [30], promotes migration/activation of microglia [29], induces iNOS, IL1β and TNFα expression, as well as the release of matrix metalloproteinase 9 and NO [31,32,33,34,35]. It is also noteworthy that, while S100B is currently regarded to be released from astrocytes with paracrine/autocrine effects, and a role for astrocytes in MS is now definitely recognized [36], its presence in oligodendrocytes [5] appears to deserve a special attention for a possible role in pathogenic MS processes.

In this study, we used PTM, an approved antiprotozoal drug [37,38] that is also known to inhibit S100B activity [11]. Because of this property, the PTM effects on RR–EAE SJL mice may be reasonably attributed to a block of S100B activity, although additional or even alternative mechanisms cannot be ruled out. It should also be noted that the inflammatory cytokines that we observed here to be affected by PTM administration are also known to be influenced by S100B [28,31,33]. Furthermore, S100B expression is reduced after PTM administration, as we have shown here in the present experimental MS animal model, also confirming what was observed in other pathological conditions where PTM exerts a protective role [39,40].

Our results thus propose that the inhibition of S100B has a significant impact on the extent of neuroinflammatory features, and this appears in line with the typical clinical and neuropathological heterogeneity of the model [41,42]. Remarkably, the expression levels of *IFNγ* and TNF*α* are strongly decreased by the PTM treatment, in line with the notion that these two cytokines play a relevant role in the CNS, in both physiological and pathological conditions [43]. Conversely, *IL-1β* expression levels are not modulated by PTM. This discrepancy is not surprising and might be ascribed to differences related to the RR–EAE model, to the specific brain areas analyzed, and, finally, to the local neuroinflammation level, parameters which are discriminative in the pathology, as also demonstrated in other EAE models [44].

All NOS isoforms are present in the CNS with a clear upregulation in reactive microglia/macrophages [45], resulting in a high generation of nitric oxide, a free radical found at higher than normal concentrations within inflammatory MS [46]. Confirming previous data, our results also show an increase in the activity of NOS in EAE mice and, furthermore, highlight that the treatment with PTM restores the activity of the enzyme to the control levels, although the effect of PTM on iNOS was already observed in vitro [47]. It still remains to be clarified the molecular mechanism(s) contributing to the reduction of NO production with the consequent anti-oxidative and anti-inflammatory potential.

As far as ROS are concerned, we have not found significant results in our experimental model. It has been widely ascertained that MS may be affected by oxidative stress. However, it was also reported that in RR–MS, the inflammatory process prevails, and oxidative stress is counteracted by antioxidant mechanisms [48]. Intriguingly, the levels of S100B protein, but not *S100B* mRNA, are reduced following PTM treatment. This might be due to a PTM action in protein synthesis steps following mRNA transcription. Alternatively, the binding of PTM to S100B might partially mask immunologically relevant epitopes of the protein.

PTM administration has also been shown to ameliorate clinical and/or neuropathologic/biomolecular parameters in other disorders involving the nervous system, such as AD, PD, sepsis-associated encephalopathy, and bowel inflammation, where its action has been reasonably attributed to the block of S100B activity [49,50,51]. In general, variations/manipulations of S100B concentration have been shown to directly correlate with clinical symptoms and/or biomolecular/pathological parameters of these disorders, which depend, as far it is known, on etiologic factors different from those hypothesized for MS, but that share with MS the occurrence of neuroinflammatory processes [5,52].

In summary, the present data identify PTM and its binding molecule S100B protein, respectively, as a novel potential drug and a therapeutic target for MS treatment. Of note, PTM is suitable for a rapid translation for clinical use, being an already-approved drug. Although PTM has limited capacity to permeate the BBB [53], and although a proportion is retained within the capillary endothelium [54], the BBB damage occurring during demyelinating diseases [55], as also shown by evidences of tracer leakage into the CNS [56], suggests that this drug may be easily available within the inflamed CNS, also when administered systemically, as in this work. Finally, the identification of S100B as a putative therapeutic target of MS pathogenetic processes might likely open novel perspectives for even more efficacious treatments.

## Supplementary Materials

The following are available online at https://www.mdpi.com/2073-4409/9/3/748/s1. Figure S1: Experimental procedure of EAE (clinical outcome and neuropathology). Table S1: Primer sequences.
