# Supplementary material for: The S100B Inhibitor Pentamidine Ameliorates Clinical Score and Neuropathology of Relapsing—Remitting Multiple Sclerosis Mouse Model
Source: Cells. 2020 Mar 18;9(3):748. doi: 10.3390/cells9030748 (PMC7140642; doi:10.3390/cells9030748)
Supplement: Supplementary file 1 [file cells-09-00748-s001.pdf]

NAME	Forward SEQUENCE	Reverse SEQUENCE
β-actin [21]	5’-CGTAAAGACCTCTATGCCAACA-3’	5’-GGAGGAGCAATGATCTTGATCT-3’
S100B	5’-AAAGGCTCATGGGCTCGAAG-3’	5’-GAAGGGGGTTGGGGTTTCAT-3’
iNOS	5’-CAGCTGGGCTGTACAAACCTT-3’	5’-CATTGGAAGTGAAGCGTTTCG-3’
TNFα[22]	5'-CGAGTGACAAGCCTGTAGCCC-3'	5'-GTCTTTGAGATCCATGCCGTTG-3'
IFNγ[23]	5’-ATGAACGCTACACACTGCATC-3’	5’-CCATCCTTTTGCCAGTTCCTC-3’
Il1β[24]	5’-TGCCACCTTTTGACAGTGATG-3’	5’-ATGTGCTGCTGCGAGATTTG -3’

Supplementary Table S1. Primer sequences.

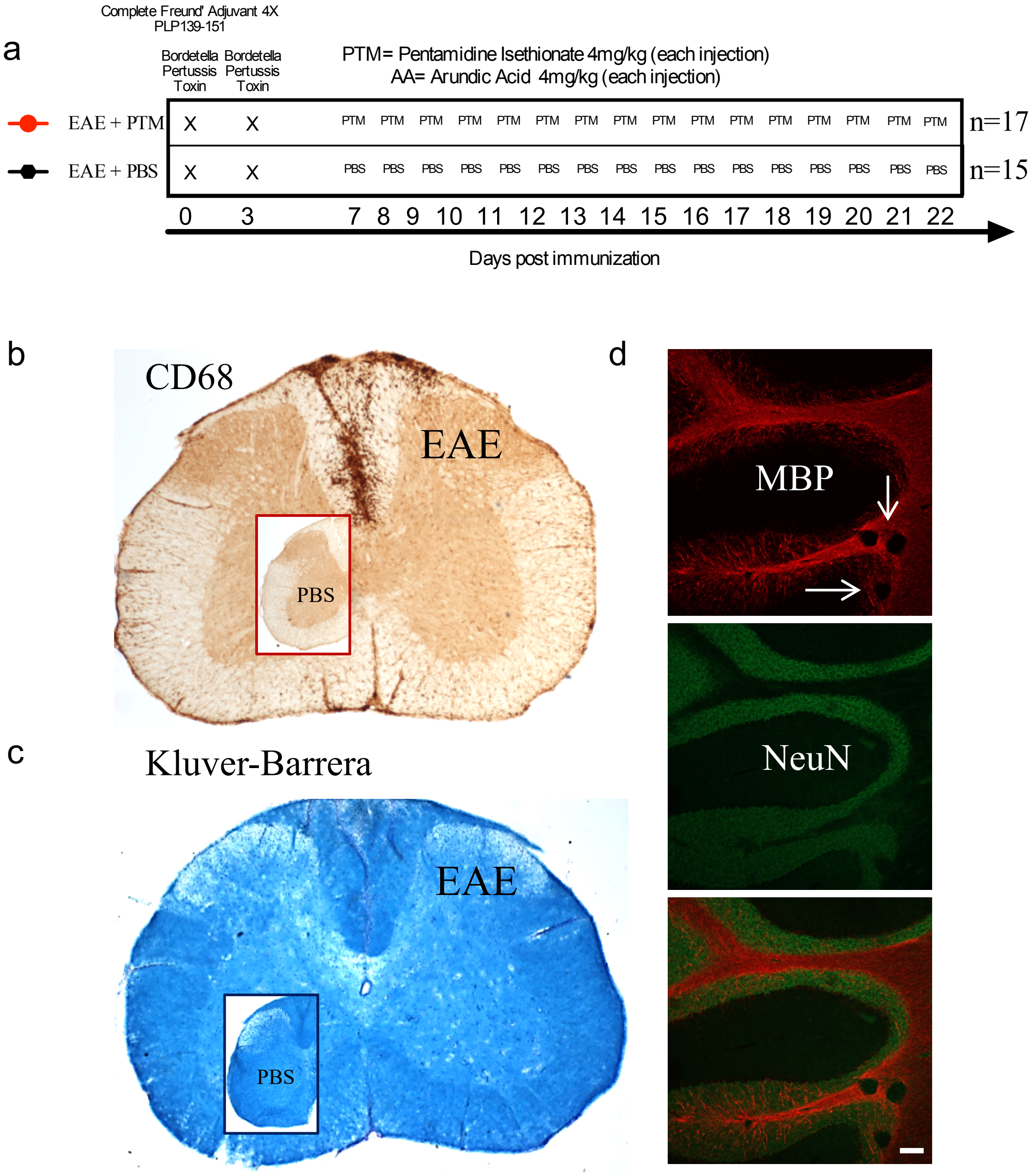

Figure S1. **Experimental procedure of EAE: clinical outcome and neuropathology.** Timeline (a) of EAE induced with PLP139-151, CFA (4X) and Pertussis Toxin, seven days after EAE induction a group of mice was treated with Pentamidine Isethionate (PTM, 4mg/kg, IP). Spinal cord sections (30 µm) from Control (**PBS**) and $PLP_{139\text{-}151}$ immunized (**EAE**) female SJL mice (8–10 weeks-old) were subjected to CD68 immunoistochemistry (b) and Kluver-Barrera staining (c), show abundant CD68-positive infiltrates and demyelinating areas. (d) As highlighted by immunofluorescence with MBP (red) and NeuN (green), cerebellar sections (30 µm) from EAE mice show clear demyelinating lesions (arrows). Scale bar = 100 µm.

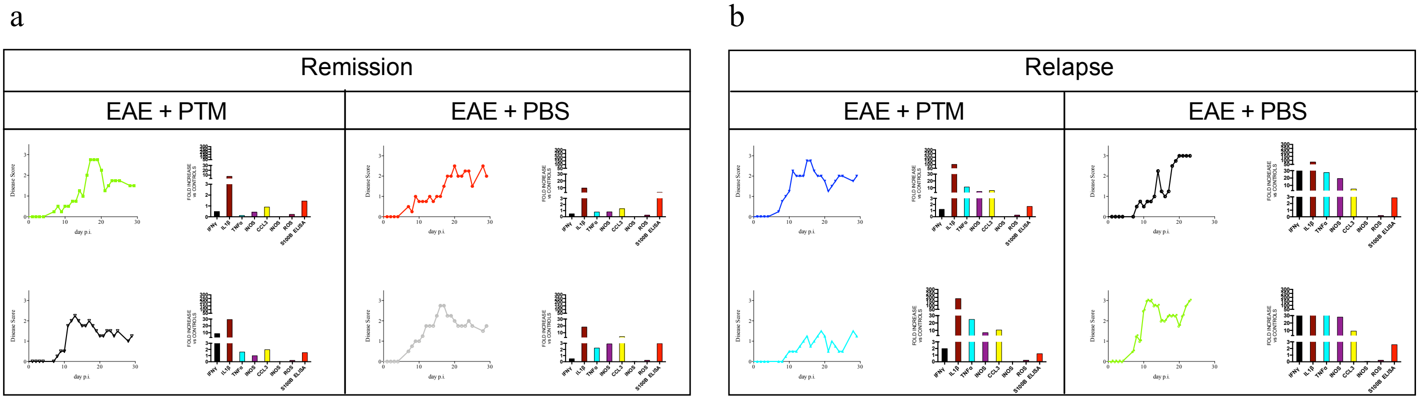

Figure S2. **Pentamidine modulates cytokines and S100B protein in the CNS of RR-EAE mice, particularly during relapse.** The gene expression levels of inflammatory cytokines are individually displayed for the different disease courses of each mouse; here we show the most interesting cases on the basis of their EAE phases. As expected, inflammatory cytokines follow disease activity (a=remission, b=relapse), but interestingly Pentamidine (PTM) reduces the expression levels of these genes particularly during the relapse**.** Here we display mice, all sacrificed 30 days after immunization and all in the relapse/late onset of EAE (score between 2 and 3): 4 untreated and 4 treated with PTM. c-f) Linear regression analysis (lower panel) comparing single cytokine expression levels in the brain (y-axis) and mean disease score of each EAE animal in both conditions: PTM-treated (red lines and symbols) and untreated (black lines and symbols). The difference of elevations or intercepts between the PTM-treated and untreated resulting slopes (F) are indicated with statistical significance (p, all significant except d).

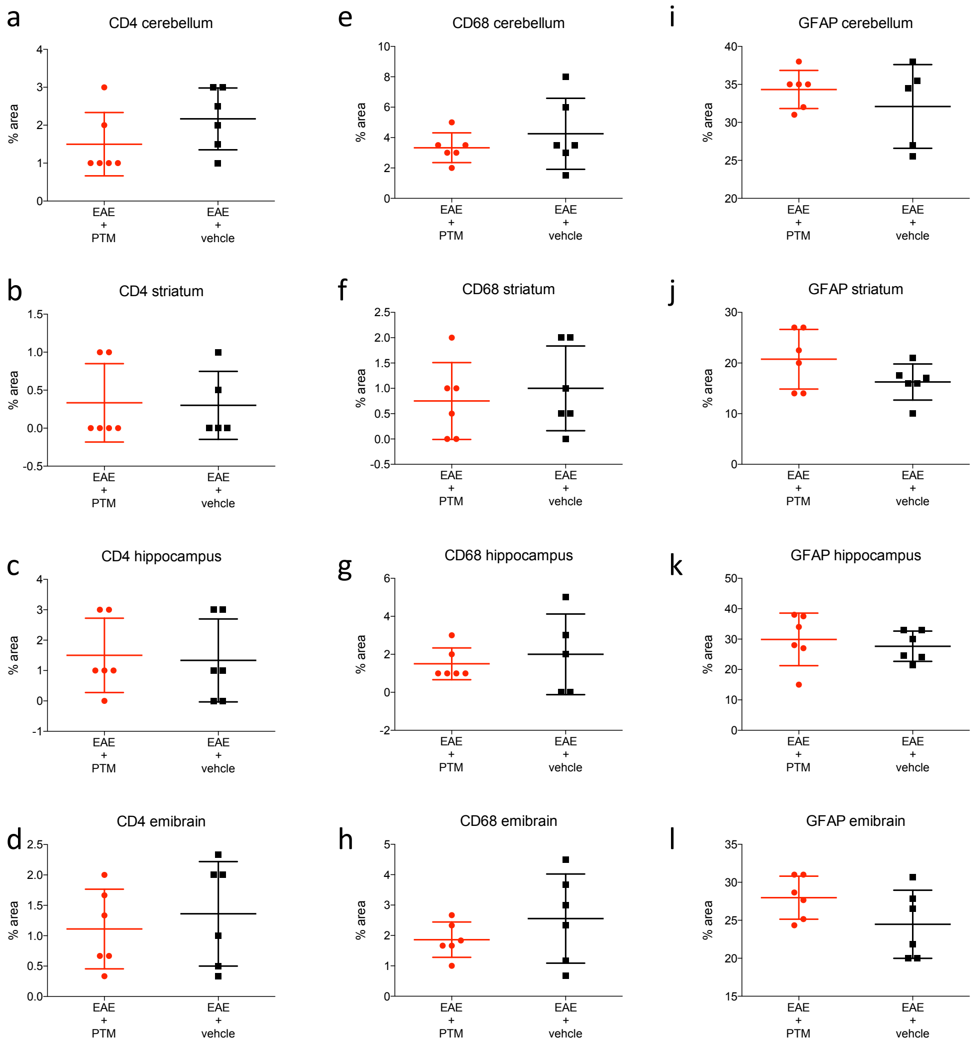

Fig S3 **Heterogenous Impact of Pentamidine in the different areas of CNS during RR-EAE in each mouse.** Immunocytochemistry and DAB assays revealed that pentamidine (PTM) seems to act on immune infiltrates but the expression of CD4 (a-d), CD68 (e-h) could be heterogenous depending on each mouse/disease activity and each analyzed area. Similar results for GFAP percentage (i-l). All these regional cell counts, although not significant, indicate a different impact of the inhibition of S100B for each area of the CNS.
